# Odontological Society of Pennsylvania

**Published:** 1895-08

**Authors:** 

**Affiliations:** Odontological Society of Pennsylvania


### ODONTOLOGICAL SOCIETY OF PENNSYLVANIA.

The regular meeting of the Odontological Society of Pennsylvania was held February 9, 1895, President C. N. Peirce in the chair. Upon the conclusion of routine business, the President called upon the essayist of the evening, Dr. R. M. Sanger, to read his paper on salol.

(For Dr. Sanger's paper, see page 472.)

### DISCUSSION.

*Dr. Crouse.*—I am not at all familiar with the material here recommended, and should not care to give an opinion without some investigation. I have had one material for root-filling that has satisfied me for twenty years. I am not casting around for new root-filling materials. I have but one way of filling a root of a tooth, and I do that with oxychloride of zinc, carrying it to the point of the root, with one or two laps of No. 10 gold on a very small broach, wrapped on in such a way that when the broach comes away the gold will stay there. I have practised that out of the mouth and in the mouth, and I do not know of a single failure. Still, I am very glad that some of the younger men, who have more time than I, are looking up the newer things.

*Dr. Faught.*—I have not used this method. Like Dr. Crouse, for the twenty years I have been practising I have never departed from one way. I fill all my canals with cotton. But I can readily understand how I might be induced to follow the modified plan of my worthy friend and use salol as directed.

Of course, those who use oxychloride of zinc never expect to take it away, never have failures, and never remove it; but I have seen a great many oxychloride fillings under most distressing conditions. I do not care to close a canal with anything that cannot be removed, and it seems this material can be removed at almost any time.

*Dr. Deane.*—Under encouragement of Dr. Kirk I took up the use of salol for the filling of root-canals. The method by which I apply the salol differs somewhat from that pursued by the essayist. After applying the dam, I cleanse the root thoroughly by the application of sodium peroxide in powder, working it down into the canals by the use of a fine broach from which the barbs have been removed; then, applying one or two drops of water, I allow it to remain until all effervescing has ceased. A piece of sponge is laid by the side of the cavity to absorb the water. I wash out all the solution of peroxide and apply hot air; when thoroughly dry, a fine broach is warmed and carried as far up the canal as possible. Then the salol is taken between the beaks of a pair of long, flat foil-pliers and passed over the alcohol flame, when it immediately melts. I then place the points of the foil-pliers, containing the melted salol, on either side of the warm broach in the root-canal, and gently open the forceps. The salol will immediately run up the broach and fill the canal. Gently withdraw the broach, and if any air is at the apex of the root it will be seen to follow the broach down and appear in a bubble on the surface of the salol. Apply cold air and fill at once.

An experience has led to some thought. Recently I filled a lower six-year molar. I felt sure both roots were entered. It was a case I could watch carefully. It was opened the other day, and in one of the roots not a particle of salol was found; and yet it had been carefully sealed. Dr. McQuillan has had the same experience.

*Dr. Sanger.*—Was the root putrescent before it was filled?

*Dr. Deane.*—Yes; and there was an abscess, I believe. The apex of the root was open sufficiently to allow the salol to pass through. The abscess healed very nicely, but I want to know what became of the salol.

I wish to call the attention of the Society to the use of sodium peroxide for cleansing putrescent canals. Take a spatula and force some of it into the root with a broach, and moisten with a little water. It will boil up and fume a few minutes; then wash it out and dry the root with heat. I have so treated several roots that had bad putrescent pulps, and filled them immediately. Up to this time I have had perfect satisfaction with the treatment.

*Dr. Broomall.*—Has salol any therapeutic action upon a diseased root, or does it act only as a sealing structure?

*Dr. Sanger.*—As stated in the paper, the action of salol is that of an antiseptic. I have thought the action of the crystal might be analogous to that of carbolic acid; whether it is so has not been determined.

*Dr. Broomall.*—That is what I wished to know. When we fill roots with cotton we usually soak it in some remedial agent. I was wondering if this article had any such qualities that would render it superior to carbolic acid.

*Dr. Curry.*—I have used salol in six cases, and in four of the six there was decided pain. Has the essayist found it to contract or crystallize?

*Dr. Sanger.*—I have no knowledge of its contraction. I have tried it in roots out of the mouth and in glass tubes, and have never had any trouble such as the doctor describes. I am at a loss to understand the cause of pain, unless the salol passed through the apical foramen.

*Dr. Brubaker.*—I believe it is the general impression that salol, when it comes in contact with the alkaline basis in the small intestines, is converted into carbolic acid or salicylic acid, and all the virtues of salol are largely attributed to these agents.

If salol is introduced into the tooth and it passes through the apical foramen, there is a possibility that the alkaline plasma which is around the foramen of the tooth would decompose the salol into carbolic acid and salicylic acid. Therefore in the root-canals salol would simply act like carbolic acid.

*Dr. Warren.*—I have never used it clinically, but have had experimented with it out of the mouth. With lower molars or bicuspids, where gravity would act, the roots, on being opened, were found to be filled perfectly to the end; but where the tooth was held in such a position as they would occupy in the upper jaw, the roots were not filled. When a warm instrument was introduced, gravity would draw the material back.

No one else responding, the President called upon Dr. Sanger to close the discussion.

*Dr. Sanger.*—I purposely avoided the question of root-treatment. We presupposed that the root was ready to fill. A perfect root-filling has not been found. One of the most scientific papers, to my mind, on the subject of root-filling, was read by a gentleman in the South, where he described a method of filling roots with paraffine, by the use of an electrode and metallic points. It pleased me because our object in filling a root is, if possible, to seal it hermetically, so that it shall not in the future become contaminated from within or without. You understand clearly the position taken by some in regard to porosity of the root. Remedies in the canal may pass out through the canaliculi, and, reasoning by analogy, if they can pass out, why can't they pass in? We do not discuss that subject, but if we can seal the canal perfectly, seal it hermetically, we are a step nearer to the desired point.

One of the beauties of this remedy, to my mind, is its easy removal. A good general always keeps his base of supply open, likewise his road of retreat, and so I think it is scientific for us to always leave a means of retreat; the more easily we can do that for the patient, the more scientific our treatment is, although such precaution is a tacit acknowledgment that we are sometimes obliged to make a retreat.

The value of salol is not alone as a root-filler; if it had been, I should have confined my paper to that subject; but, for the uses I have described, I think it is well worthy of experiment.

*Dr. Bonwill.*—Ever since the implantation of the roots of teeth I have been interested in that method, and have been experimenting in a parallel line. What I shall speak of is the feasibility of implanting metallic tubes into the alveolar process from three-eighths to half an inch in depth. The object of the appliance is to take the place of bridge-work.

He then exhibited a model that consisted of a tube into which was inserted a pin that could support a crown; the pin moved easily in and out of the tube.

JOSEPH HEAD, M.D., D.D.S.,
*Editor Odontological Society of Pennsylvania.*

